# The effect of the addition of propolis to resin-modified glass ionomer cement bracket adhesive materials on the growth inhibition zone of *Streptococcus mutans*

**DOI:** 10.12688/f1000research.20717.2

**Published:** 2020-07-27

**Authors:** Stefani Kristanti Saputra, Darmawan Sutantyo, Cendrawasih Andusyana Farmasyanti, Ananto Ali Alhasyimi

**Affiliations:** 1 Department of Orthodontic, Faculty of Dentistry, Universitas Gadjah Mada, Yogyakarta, Indonesia

**Keywords:** Propolis, Resin Modified Glass Ionomer Cement, Inhibition Zone, Streptococcus mutans.

## Abstract

**Background:** Orthodontic treatments progress alongside the development of adhesive materials. The present study aimed to determine the antibacterial properties of propolis, a natural product, in a mixture of resin-modified glass ionomer cement by observing the growth inhibition zone of *Streptococcus mutans.*

**Methods: **This was an *in vitro* study conducted on 45 samples of adhesive material, which were divided into control group (resin-modified glass ionomer cement bracket adhesive material), two groups of propolis concentrations (15%, and 25%) and duration (0, 15, and 30 days). The antibacterial effect of each sample was evaluated against *S. mutans* using an agar plate diffusion test. Measurement of the diameter of the growth inhibition zone of *S. mutans* was carried out. The data obtained were analyzed statisticallyThe significance value of the Kruskal Wallis and Mann-Whitney test was set at p <0.05, while the significance value of the normality and homogeneity test was set at p> 0.05). Datas in this study that were not normally distributed (p = 0.012) but homogeneous (p = 0.110) were analyzed by the Kruskal-Wallis test (p = 0.003) and then the Mann-Whitney test was performed to determine differences in significance between groups.

**Results:** There was a relationship between concentration and duration of propolis to the growth inhibition zone of *S. mutans* (p=0.003). The addition of 25% propolis concentration inhibited the growth of *S. mutans* more than the addition of 15% and without propolis (control group). The addition of propolis to resin-modified glass ionomer cement for 15 days was more effective in inhibiting the growth of *S. mutans.*

**Conclusion: **The addition of propolis to adhesive materials provides an inhibitory effect on the growth of *S. mutans*, which may be effective in the world of preventive dentistry.

## Introduction

Fixed orthodontic treatment can be a risk factor for plaque accumulation ^1^, which is significantly affected by the presence of bracket attachments and archwires ^2^. A large number of areas for microbial colonization during orthodontic treatment can cause plaque accumulation, especially around the bracket and cervical edge of the band ^3^. As many as 60% of fixed orthodontic patients also show poor oral health, which is shown by the high plaque index value during orthodontic treatment and the presence of white spot lesions in ~50% of orthodontic patients ^4, 5^. A study also reported that the incidence of fixed orthodontic patients with one new white spot lesion during treatment was 72.9% and the incidence of cavity lesions was 2.3% ^6^.

The presence of fixed orthodontic appliances in the oral cavity increases microbial population. Observational studies have shown that there is a positive relationship between the use of fixed orthodontic appliances and the number of bacteria, such as *Streptococcus mutans,* on plaques, which is known as bacteria in early or initial caries ^7– 9^. These bacteria can attach to all surface locations in the oral cavity including the surface of the bracket and the area adjacent to the bracket ^10^. Efforts to protect areas that are vulnerable to bacterial colonization needs to be done, especially the area around the bracket.

Fixed orthodontic treatment develops fast along with the development of adhesive materials used to attach brackets to the tooth surface ^11^. Brackets are attached to the teeth using acid etching or cemented using glass ionomer cement ^8^. Resin-modified glass ionomer cement is developed by adding hydrophilic resins such as hydroxydimethacrylate and BIS-GMA to conventional glass ionomer cement. This material is not only known to be attached to the surface of the tooth, but is also able to release fluoride, has better physical properties, shorter setting time, and is more effective against humidity ^12^. At present, new research has been performed to develop a dental material with antibacterial activity, which will play an important role in preventing caries. Glass ionomer cement as bracket adhesive material can release fluoride but the desired antibacterial effect needs to be enhanced ^13^.

Over the past few decades, the use of natural products for pharmacological purposes has increased in the world ^14^. Propolis is a sticky resin substance collected by honey bees from the sap of plants, leaves, and buds, which are mixed with the sap and saliva of bees in the nest. There are more than 180 chemical substances contained in propolis and are influenced by the type of bee, climate, plants and trees, and the time of collection. Bees use propolis to strengthen the nest wall and protect it from infection, and the human population uses this product for many purposes ^15^, for example, propolis is known to provide protection against cariogenic bacteria and oral pathogens ^16^. Hasan *et al.* ^17^, stated that there are few studies about propolis activity from Indonesia.

Propolis, as a natural product, can be used in a mixture of glass ionomer cement in order to increase antibacterial activity ^13, 18^. Megawati ^19^ investigated the shear bond strength of metal brackets bonding to resin-modified glass ionomer cement adhesive materials combined with 25% and 50% propolis. The addition of 50% propolis concentration had better shear bond strength and was able to withstand shear bond strength of 6–8 MPa according to the standard strength of clinically acceptable adhesive materials. The antibacterial potential of propolis added to resin-modified glass ionomer cement needs to be further investigated because orthodontic treatment takes place over a long period of time. Hatunoglu *et al.* ^13^ has also investigated that the addition of 25% and 50% Turkish propolis to glass ionomer cements increased antibacterial activity without modifying the mechanical properties of glass ionomer cements ^13^ but they have not conducted time-related research. Further research on the effect of the addition of15%, and 25% Indonesian propolis for 0, 15 and 30 days on resin-modified glass ionomer cement to the growth inhibition zone of *S. mutans* has never been done. Therefore, our research was the first study to investigate the relationship between concentration and duration of propolis to the growth inhibition zone of *S.mutans*.

 The present study was an *in vitro* experimental study done in order to determine the antibacterial activity of resin-modified glass ionomer cement combined with Indonesian propolis on *S. mutans*. The hypothesis was there was a relationship between concentration and duration of propolis to the growth inhibition zone of *S.mutans.* Adding propolis to this orthodontic bracket adhesive will increase the antibacterial properties of the adhesive.

## Methods

Ethical clearance for the study was obtained from Faculty of Dentistry, Universitas Gadjah Mada, Yogyakarta, Indonesia (No.001621/KKEP/FKG-UGM/EC/2018).

### Preparation of propolis extract

**Figure 1.**
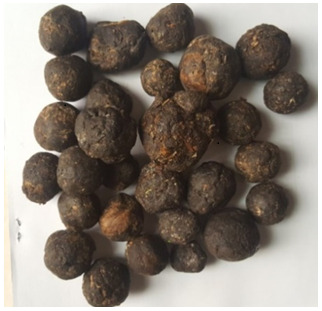
Propolis produced by *Apis mellifera*.

Pure propolis was produced by honeybees ( *Apis mellifera*) in Indonesia ( Figure 1). The propolis (970g) was purchased from Klinik Apitheraphy Kusuma (Moyudan, Sleman, Daerah Istimewa Yogyakarta) and the production of the extract was done in LPPT Unit 1, Universitas Gadjah Mada, Yogyakarta, Indonesia. Propolis samples were chopped into small pieces and ground using a blender. Then, each 250 g sample of propolis was dissolved in 2500 mL of ethanol 80%, stirred at 800 rpm for 30 minutes using an electric stirrer (RW 20 digital; IKA, Germany) and left for 24 hours at room temperature. Rough particles were removed from the propolis extract using rough filter paper (58 cm × 58 cm) and the propolis was stirred once again for 30 minutes using an electric stirrer *,* left for 24 hours, and filtered. The filtrate was concentrated by a vacuum rotary evaporator. Next, the extract is poured in a porcelain cup and heated with a waterbath (70 °C) to produce propolis extract. Samples were kept in a dry and dark place until they were used ^19^.

### Preparation of resin-modified glass ionomer cement containing propolis

**Figure 2.**
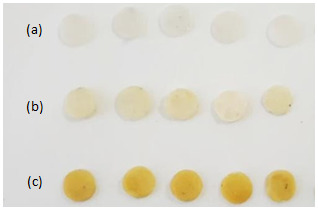
Adhesive materials ready to be tested. Resin-modified glass ionomer cement ( **a**) control, ( **b**) 15% propolis, and ( **c**) 25% propolis.

Conventional adhesive resin-modified glass ionomer cement (Fuji Ortho LC, GC, Japan), which is made up of powder and liquid, was used in this study. Samples were prepared containing the conventional resin-modified glass ionomer cement liquid and the two concentrations of propolis (15% and 25%) : (i) Resin-modified glass ionomer cement without propolis (Powder ^RMGIC^:Liquid ^RMGIC^ ratio = 1:1) (n=15); (ii) resin-modified glass ionomer cement with 15% propolis (Powder ^RMGIC^: Liquid ^RMGIC^:Propolis Extract ratio = 1:0.85:0.15) (n=15); (iii) resin-modified glass ionomer cement with 25% propolis (Powder ^RMGIC^: Liquid ^RMGIC^:Propolis Extract ratio = 1:0.75:0.25) (n=15). Each group of samples was incubated for 0, 15, and 30 days (n=5/time duration). The adhesive materials were mixed according to the manufacturer’s instructions. After mixing the powder and liquid of each cement, samples were put into cylindrical molds (5 mm in diameter and 0.64 mm thickness), and the upper surface was flattened by pressing down and exposed by light-curing unit (LY-B200, Liang Ya, China) for 10 seconds each surface ( Figure 2) ^19, 20^.

### Agar disk diffusion test

**Figure 3.**
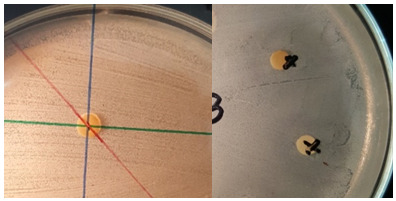
Measurement of growth inhibition zone diameter. Left panel, measurement guidance; right panel, growth inhibition zone of *S. mutans.*.

Agar disk diffusion test was performed at the Microbiology Laboratory, Faculty of Veterinary, Universitas Gadjah Mada, Indonesia. *S. mutans* ATCC 25175 type strain was used throughout the study. It was pure culture from the isolated laboratory of the Microbiology, Faculty of Veterinary, UGM, Yogyakarta, Indonesia. Bacterial strain from stock cultures was cultivated in Brain Heart Infusion broth at 37°C for 24 hours, corresponding to 10 ^8^ CFU/mL using the McFarland scale. *S. mutans* was spread uniformly on the surface of Mueller Hinton Agar plates to produce a lawn. Adhesive samples were inserted in the plates. After a 24h incubation period in an incubator at 37°C, the plates were taken out of the incubator and the antibacterial activity was evaluated using a digital caliper to measure the diameter of halos of growth inhibition of the strain at three different points. The inhibitory zone was considered the distance (mm) from the outside margin of the samples to the initial point of the microbial growth ( Figure 3). The mean was calculated for each sample and all measurements were performed by the same blinded operator ^21^.

### Statistical analysis

Data were analyzed using SPSS IBM for Windows (Version 22.0). The normality and homogeneity of variance in each group was confirmed before analyzing the data. Saphiro-Wilk normality test is conducted to determine the distribution of data and the Levene homogeneity test is carried out to determine the homogeneity of data variance. Datas of resin-modified glass ionomer cement without propolis, 15%, and 25% propolis groups for 0, 15, and 30 days group were not normal (p = 0.012) but homogenous (p = 0.110), so they were analyzed using Kruskal Wallis test and then Mann-Whitney test to determine the significance differences between groups. The significance level was set at 5%. The significance value of the normality and homogeneity test was (p> 0.05) while the significance value of the Kruskal Wallis and Mann-Whitney test was (p <0.05).

## Results

**Figure 4.**
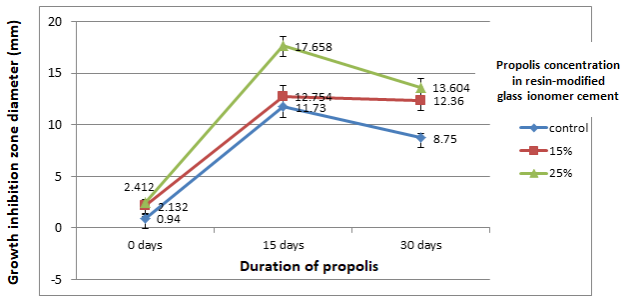
Means and standard deviation of growth inhibition zone of *Streptococcus mutans* among groups.

Propolis, as a natural product, was combined with resin-modified glass ionomer cement in order to assess antibacterial activity against *S. mutans*. The mean diameters of bacterial growth inhibited by different concentrations and duration of propolis combination in the adhesive are shown in Table 1 and Figure 4. The growth inhibition zone in the present study was shown as a transparent or clear area around the adhesive materials.

**Table 1.** Means and standard deviation of growth inhibition zone of *Streptococcus mutans* among groups. Percentages are propolis concentration in resin-modified glass ionomer cement.

Adhesive materials	Duration (days)	n	Mean (mm)	Standard deviation
Resin-modified glass ionomer cement (control)	0	5	0,940	0.370
	15	5	11,730	0.720
	30	5	8,75	0.359
Resin-modified glass ionomer cement with 15% propolis	0	5	2.132	0.293
	15	5	12.754	1.053
	30	5	12.360	0.359
Resin-modified glass ionomer cement with 25% propolis	0	5	2.412	0.328
	15	5	17.658	0.928
	30	5	13.604	0.892

Table 1 and Figure 4 provides summarized data regarding the agar diffusion method and show the mean and standard deviation values of the diameter of growth inhibition zone for each sample in the groups against the *S.mutans* strain. Clear inhibition zones were shown showing that the propolis enhanced the antibacterial effect of the resin-modified glass ionomer cement. Among the concentrations (control, 15%, and 25% propolis), control group still showed inhibitory effect reflecting that the resin-modified glass ionomer cement has its own antibacterial activity. The addition of 25% propolis revealed higher antibacterial activity than 15% and control ( Table 1 and Figure 4). As can be seen in Table 1 and Figure 4, the mean diameter of growth inhibition zone for 0 days was lower than 30 and 15 days. In general, the duration of propolis after 15 days resulted in a greater inhibition zones compared with 30 and 0 days.

**Figure 5.**
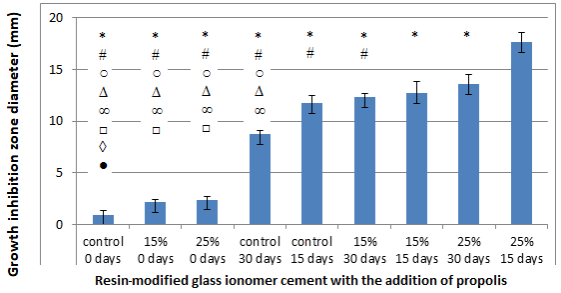
The graph of Mann-Whitney test result between adhesive materials. Note : Statistically significant (p<0.05). * = < 25% 15 days, # = < 25% 30 days, ○ = < 15% 15 days, ∆ = < 15% 30 days, ∞ = < control 15 days, □ = < control 30 days, ◊ = < 25% 0 days, ● = < 15% 0 days

Looking at the data together (concentration and duration, it was observed that there was an interaction between concentration and duration of propolis to the growth inhibition zone against *S.mutans* ( Table 2 and Figure 5). The antibacterial activity of resin-modified glass ionomer cement with 25% propolis for 15 days was the highest among all other concentrations for all tested days (p<0.05).

**Table 2.** Mann-Whitney test results between adhesive materials. *Statistically significant (p<0.05)

Group	IA	IB	IC	IIA	IIB	IIC	IIIA	IIIB	IIIC
IA		0.009 *	0.009 *	0.009 *	0.009 *	0.009 *	0.009 *	0.009 *	0.009 *
IB			0.009 *	0.009 *	0.175	0.117	0.009 *	0.009 *	0.009 *
IC				0.009 *	0.009 *	0.009 *	0.009 *	0.009 *	0.009 *
IIA					0.009 *	0.009 *	0.347	0.009 *	0.009 *
IIB						0.917	0.009 *	0.009 *	0.076
IIC							0.009 *	0.009 *	0.016 *
IIIA								0.009 *	0.009 *
IIIB									0.009 *
IIIC									

I: Resin-modified glass ionomer cement; II: Resin-modified glass ionomer cement with 15% propolis ; III: Resin-modified glass ionomer cement with 25% propolis; A: 0 days; B: 15 days; C: 30 days

## Discussion

An increase in the number of bacteria and plaques in the oral cavity of fixed orthodontic patients is one challenge for orthodontists. *Streptococcus mutans* is the first bacteria that play an important role in the etiology of caries because this species form a colony and be the initiator of other bacteria to do the same thing ^22^. In the present study, a growth inhibition zone as a high concentration of propolis increased the antibacterial activity of the adhesive material to *S.mutans,* which was shown by an increase in the diameter of the growth inhibition zone. Resin-modified glass ionomer cement with the addition of 25% propolis showed the largest diameter of the inhibition zone in all periods, indicating the highest antibacterial activity compared to other treatment groups (15% propolis and control group). Therefore, the addition of propolis to orthodontic adhesive materials as an antibacterial agent to inhibit the growth of *Streptococcus mutans* can be considered. This is in accordance with Asdar *et al.* ^22^, who stated that propolis could inhibit the growth of *S. mutans* and it appeared in diameter changes of the inhibition zone. The greater the concentration of propolis, the greater the effect of inhibition produced.

The results agree with many researchers who have demonstrated the antibacterial activity of propolis. The mechanism of propolis against microorganisms is complex. Propolis works by inhibiting bacterial mobility and enzyme activity, as well as affecting the cytoplasmic membrane, which changed membrane permeability ^15^. The functional and structural damage is suspected to occur due to components in propolis extract, such as flavonoids as the main constituent (quercetin, galangin, and pinocembrin), caffeic acid, benzoic acid, and cinnamic acid ^17, 23, 24^. Koo *et al.* ^25^, revealed that flavonoids, the largest component of propolis *Apis mellifera*, works by inhibiting glycosyltransferase activity. Extracellular polysaccharides, generally glucans, are produced by glucosyltransferase and play a role in the cariogenicity of dental biofilms ^26^. Therefore, inhibition of glucosyltransferase interferes with cell metabolism through biochemical reactions and has the potential to prevent caries, especially in fixed orthodontic patients. Flavonoids interact with bacterial cell walls, forming complex compounds with extracellular proteins through hydrogen bonds so that they inhibit the activity of microorganisms, including bacterial mobility ^27^. In addition, Pelczar and Chan ^28^ showed that flavonoids denature and coagulate bacterial cell proteins so that cell damage cannot be repaired. Flavonoids could penetrate bacterial cell peptidoglycans so that the cell layer is not intact. The instability of cell walls causes the permeability of the cell and the control of the protein composition to be disrupted so that bacterial cells lose their shape and are lysed ^28^. Pelczar and Chan ^28^ also revealed that the higher the concentration of an antibacterial agent the stronger the antibacterial activity. An increased concentration of propolis added to the resin-modified glass ionomer cement in the present study resulted in a darker color. The antibacterial ability of the resin-modified glass ionomer cement increased with a higher propolis concentration. The results of the study were also in line with research conducted by Woo ^29^, who concluded that the addition of antibacterial agents to glass ionomer cement, such as propolis, which had a darker color of propolis indicated more flavonoid as an active substance; therefore, the antibacterial activity increased with higher flavonoid content.

The present results showed that the duration of propolis treatment significantly affected the growth inhibition zone of *S.mutans* on resin-modified glass ionomer cement. There were smaller diameter values of the growth inhibition zone on all types of adhesive materials with the addition of propolis for 30 days compared with 15 days which indicated lower antibacterial activity of the adhesive material. This is similar to research that examined the antibacterial effect of resin-modified glass ionomer cement and concluded that there was an increase in the average diameter of inhibition zones over time, especially in the first week, namely days 1, 3 and 7 ^30^.

Higher antibacterial activity on day 15 than day 0 in the present study could be caused by several things, such as changes in pH and release of fluoride. An inhibition zone was suspected to be caused by the production of a low pH around the test material. The resin-modified glass ionomer cement liquid component contained hydroxylethyl methacrylate (HEMA), which may have facilitated low pH and contributed to antibacterial properties ^31^. Prasad and Maradia ^32^ also added that the initial value of pH after mixing was also acid, where most bacterial growth would be suppressed then the pH value began to increase to a neutral level where it was not enough to inhibit bacterial growth. Kavita *et al.* ^33^, mentions that an increase in pH and a decrease in the release of fluoride ions explains the decrease in antibacterial activity.

Fluoride inhibits the acid production and glucans by *S. mutans*, which has been stated by Wiegand *et al.* ^34^, who reviewed that the release of fluoride could reduce demineralization, increase remineralization, and inhibit bacterial growth so that glass ionomer cement was cariostatic. Additionally, Featherstone ^35^ demonstrated that fluoride worked by inhibiting bacterial metabolism through changes in hydroxyapatite on enamel to fluorapatite so that the enamel was more resistant to acid and increased remineralization. The high release of fluoride in resin-modified glass ionomer cement occurred because the acid-base reaction was slowed down by the resin component, causing the ionized matrix to be less mature and capable of releasing more fluoride when compared to other materials, such as composite resins; greater pore size and porosity in resin-modified glass ionomer cement; lower solubility and higher proportion of liquid powder with high viscosity ^36^. Material with a resin content that is slightly like resin-modified glass ionomer cement has a higher porosity, which facilitates the diffusion of fluoride ^37^. This is also in line with research of Fucio *et al.* ^38^, who found that resin-modified glass ionomer cement changed in fluoride ion release over time. In that study, the release of fluoride at the beginning of the study occurred because the glass particles reacted with polyalkenoic acid, while continuous fluoride release could be caused by the ability of fluoride to diffuse through the cement pore. According to Toba *et al.* ^39^, the hydrophilic property of HEMA from resin-modified glass ionomer cement is required for the water absorption process and helps the diffusion of fluoride, which causes an increase in the release of fluoride ions.

In the present study, a smaller diameter of the inhibition zone on day 30 than day 15 indicated that the antibacterial effect of the material decreased over time. The result of this study was in accordance with Matalon *et al.* ^40^, who showed that high antibacterial potency of resin-modified glass ionomer cement at the beginning of their study decreased for the next 3 weeks even though the antibacterial material was durable. Therefore, fluoride release of resin-modified glass ionomer cement could decrease significantly with long-term use ^41^. The antibacterial activity of resin-modified glass ionomer cement in the present study was lower on the 30th day.

Statistical analysis showed that there was an interaction between concentration and duration of propolis to the growth inhibition zone of *S. mutans* (p<0.05). This means that our hypothesis was accepted. The results of the study were following the study by Dastjerdie *et al.* ^5^, where the antibacterial activity of adhesive materials to the growth of *S. mutans* depended on the type of cement and time.

Diameter of the inhibition zone in the present study was smaller on day 30 compared with day 15 with the addition of propolis; however, this is classified as a strong response (11–20 mm) compared to the response of resin-modified glass ionomer cement without addition of propolis, classified as a medium response (5–10 mm) ^42^. A survey of 500 patients at a public health center in Jakarta and dental hospital at Universitas Indonesia conducted by Maringka and Herda ^43^ showed that 90% of respondents had experienced bracket detachment and around 60% of respondents experienced this event before their next appointment (three weeks after placement). Therefore, a strong response from the results of the day 30 treatment would be enough in the first 3 weeks. Although resin-modified glass ionomer cement as an orthodontic adhesive material that releases fluoride has been used, the addition of propolis to orthodontic adhesive materials also provides an additional effect on the growth of *S. mutans* and was quite effective in this study. Propolis, as an antibacterial additive to resin-modified glass ionomer cement, still needs further research aimed at getting a combination of physical properties, propolis concentration, shear strength, and tensile strength that meet the optimal standard of orthodontic adhesive; increasing stability and adequating propolis resistance.

## Conclusion

The addition of propolis to adhesive materials gives an inhibitory effect on the growth of *Streptococcus mutans*. There was an interaction between concentration and duration of propolis and antibacterial effect against *Streptococcus mutans*.

## Data availability

### Underlying data

Figshare: Growth Inhibition Zone Around Resin-modified Glass Ionomer Cement Bracket Adhesive, https://doi.org/10.6084/m9.figshare.10263275.v4 ^44^.

This project contains the following underlying data:

- Growth inhibition zone around resin-modified glass ionomer cement bracket adhesive

Data are available under the terms of the Creative Commons Zero “No rights reserved” data waiver (CC0 1.0 Public domain dedication).
