# Supplementary material for: The effect of the addition of propolis to resin-modified glass ionomer cement bracket adhesive materials on the growth inhibition zone of Streptococcus mutans
Source: F1000Res. 2020 Jul 27;8:2105. Originally published 2019 Dec 16. [Version 2] doi: 10.12688/f1000research.20717.2 (PMC7388193; doi:10.12688/f1000research.20717.2)
Supplement: Peer review history

# Peer review: Reviewer response for version 2

**DOI:** 10.5256/f1000research.28168.r68028

**Published:** 2020-07-28

**Authors:** Ida Bagus Narmada, Alexander Patera Nugraha

**Affiliations:** 1 Department of Orthodontics, Faculty of Dentistry, Airlangga University, Surabaya, Indonesia; 2 Faculty of Medicine, Airlangga University, Surabaya, Indonesia

The authors have revised the manuscript successfully as per our suggestion. In addition, we strongly recommend this paper.

Is the work clearly and accurately presented and does it cite the current literature?

Yes

If applicable, is the statistical analysis and its interpretation appropriate?

I cannot comment. A qualified statistician is required.

Are all the source data underlying the results available to ensure full reproducibility?

Yes

Is the study design appropriate and is the work technically sound?

Yes

Are the conclusions drawn adequately supported by the results?

Yes

Are sufficient details of methods and analysis provided to allow replication by others?

Yes

Reviewer Expertise:

Orthodontics

We confirm that we have read this submission and believe that we have an appropriate level of expertise to confirm that it is of an acceptable scientific standard.

# Peer review: Reviewer response for version 1

**DOI:** 10.5256/f1000research.22785.r61777

**Published:** 2020-04-08

**Authors:** Lidia Audrey Rocha Valadas

**Affiliations:** 1 Postgraduate Program in Development and Technological Innovation in Drugs, School of Pharmacy, Federal University of Ceara, Fortaleza, Brazil

Introduction:

- You should justify the reason for studying just *S.mutans*, why not more species related to orthodontics fixed appliances?

Materials and methods:

- You should cite the georeferencing of the extract and the chemical analysis performed to identify the main constituents.
- I don’t agree with the statement about 3 groups with different concentrations - I suggest 2 groups and one control. 0% is not a propolis concentration group.

Discussion:

- Add the limitations of the study.
- Please check reference 34, it is a review, in your discussion you cited it as a research article.

Is the work clearly and accurately presented and does it cite the current literature?

Yes

If applicable, is the statistical analysis and its interpretation appropriate?

Yes

Are all the source data underlying the results available to ensure full reproducibility?

Yes

Is the study design appropriate and is the work technically sound?

Yes

Are the conclusions drawn adequately supported by the results?

Yes

Are sufficient details of methods and analysis provided to allow replication by others?

Yes

Reviewer Expertise:

Cariology; Natural Products

I confirm that I have read this submission and believe that I have an appropriate level of expertise to confirm that it is of an acceptable scientific standard.

## References

[1] : Clinical and Microbiological Evaluation of Brazilian Red Propolis Containing-Dentifrice in Orthodontic Patients: A Randomized Clinical Trial. *Evid Based Complement Alternat Med*.2020;2020: 10.1155/2020/8532701 8532701 10.1155/2020/8532701 32063987PMC6996680

# Response

**Published:** 2020-04-14

**Authors:** Stefani Kristanti Saputra

**Affiliations:** Dental Specialist Education Programme (Major Orthodontic), Department of Orthodontic, Faculty of Dentistry, Universitas Gadjah Mada, Indonesia

Dear Dr Lidia Valadas,

Thank you for your kind assistance in reviewing our manuscript and providing us with valuable advice for the manuscript and our future work. Please allow us to comment as follows:

**Introduction:**

**You should justify the reason for studying just *S.mutans*, why not more species related to orthodontics fixed appliances?**

The presence of fixed appliances in the oral cavity of orthodontic patients increases the microbial population, such as *Streptococcus mutans* known as bacteria that plays an important role in the etiology of caries. *S. mutans* is the first bacteria to form a colony and be the initiator of other bacteria to do the same thing. ^22^

**Material and Methods:**

**1. You should cite the georeferencing of the extract and the chemical analysis performed to identify the main constituents.**

The propolis was purchased from Klinik Apitheraphy Kusuma (Moyudan, Sleman, Daerah Istimewa Yogyakarta, Indonesia) and the production of the extract was done in LPPT Unit 1, Universitas Gadjah Mada, Yogyakarta, Indonesia. The pure propolis was produced by honeybees ( *Apis mellifera*) and  the main constituent of propolis *Apis mellifera *is flavonoid.

**2.** **I don’t agree with the statement about 3 groups with different concentrations - I suggest 2 groups and one control. 0% is not a propolis concentration group.**

We thank you for this valuable insight and it will be considered in our revision.

**Discussion:**

**1. Add the limitations of the study.**

Propolis is sufficient to be used as an antibacterial additive to resin modified glass ionomer cement, but it still needs further research aimed at the following:

a. get a combination of physical properties, propolis concentration, shear strength, and tensile strength that meet the optimal standard of orthodontic adhesive; and

b. increase stability and adequate propolis resistance.

**2**. **Please check reference 34, it is a review, in your discussion you cited it as a research article.**

Yes, it is a literature review. The purpose of the article was to review the fluoride release and recharge capabilities, and antibacterial properties, of fluoride-releasing dental restoratives, and discuss the current status concerning the prevention or inhibition of caries development and progression. We will revise and update the discussion section.

We thank you again for the valuable insight.

# Peer review: Reviewer response for version 1

**DOI:** 10.5256/f1000research.22785.r60534

**Published:** 2020-03-23

**Authors:** Ida Bagus Narmada, Alexander Patera Nugraha

**Affiliations:** 1 Department of Orthodontics, Faculty of Dentistry, Airlangga University, Surabaya, Indonesia; 2 Faculty of Medicine, Airlangga University, Surabaya, Indonesia

First of all, please allow me to congratulate the authors for attempting to undertake this study which I found quite interesting and useful as a reference for further study in the orthodontics material field. The manuscript itself is well-written and well-structured and I have to also commend the authors for this matter.

 However, I may require some clarifications on the following issues:

1. The paper is well organized and easy to follow.
2. To improve the readability, it is recommended that the text is checked by a native English speaking person as many of the sentences might be misunderstood. I suggest a revision of the grammar structures by an expert editor in revising manuscripts.

Abstract:

1. Please add the p-value used for the Kruskal Wallis test (p<0.05 or p<0.01) - add homogeneity and normality test p-value used (p>0.05) in the abstract section.
2. Please add the exact p-value obtained after the statistic analysis (p=.....).
3. Please check the keywords according to the MESH NCBI data base and sort alphabetically.

Introduction:

1. Please add your hypothesis.
2. Please add a statement or sentence about whether there are any similar studies that have been done before or not? Or is your study the first study to investigate this topic?
3. Please add the critical or important issue within this study.
4. Please add the reason why only *S. mutans* was examined in this study.

Material and Methods:

1. It would better if you add the detailed protocol or cite the protocol reference.
2. It would better if you add the confirmation of *S. mutans* colony or the detail of ATCC.
3. It would be better if you add more specific methods than agar disk diffusion test to examine the combination of propolis and GIC inhibit the *S. mutans*.
4. In this study, the authors performed the Kruskal wallis test. It would be better if the authors also described the Levene's and Shapiro-Wilk test.

Results:

1. It would be better to understand and attract the reader if you present the data in the diagrams.
2. Please add the results of the Levene's and Shapiro-Wilk tests
  .
3. Please indicate whether the difference is significant or not in your graph/diagrams as well, using some symbols (asterisk).

Discussion:

1. Discussion of the results is quite comprehensive. In analyzing the results, the authors also show citations from the previous study to support the explanation of these results.
2. The answer to the hypothesis of this study has been included at the beginning of the discussion section.
3. Please mention the limitation(s) of this study in the discussion section.

References:

- The supporting references are inadequate, please add the newest references that support this study.

Is the work clearly and accurately presented and does it cite the current literature?

Yes

If applicable, is the statistical analysis and its interpretation appropriate?

I cannot comment. A qualified statistician is required.

Are all the source data underlying the results available to ensure full reproducibility?

Yes

Is the study design appropriate and is the work technically sound?

Yes

Are the conclusions drawn adequately supported by the results?

Yes

Are sufficient details of methods and analysis provided to allow replication by others?

Yes

Reviewer Expertise:

Herbal in Orthodontics

We confirm that we have read this submission and believe that we have an appropriate level of expertise to confirm that it is of an acceptable scientific standard, however we have significant reservations, as outlined above.

# Response

**Published:** 2020-04-02

**Authors:** Stefani Kristanti Saputra

**Affiliations:** Dental Specialist Education Programme (Major Orthodontic), Department of Orthodontic, Faculty of Dentistry, Universitas Gadjah Mada, Indonesia

Dear Prof Ida Bagus Narmada & Dr Alexander Patera Nugraha,

Thank you for your kind assistance in reviewing our manuscript and providing us with valuable advices. Please allow us to comment as follows:

**Abstract:**

**1. Please add the p-value used for the Kruskal Wallis test (p<0.05 or p<0.01) - add homogeneity and normality test p-value used (p>0.05) in the abstract section.**

**2.** **Please add the exact p-value obtained after the statistic analysis (p=.....).**

The significance value of the normality and homogeneity test was (p> 0.05) while the significance value of the Kruskal Wallis test was (p <0.05).

Datas in this study that were not normally distributed (p = 0.012) but homogeneous (p = 0.110) were analyzed by the Kruskal-Wallis test (p = 0.003) and then the Mann-Whitney test was performed to determine differences in significance between treatment groups. The significance value of the Mann-Whitney test was (p<0.05).

**3. Please check the keywords according to the MESH NCBI data base and sort alphabetically.**

Disk Diffusion Antimicrobial Tests. Glass Ionomer Cement. Propolis. Streptococcus mutans.

**Introduction:**

**1. Please add your hypothesis.**

There is an relationship between concentration and duration of propolis to the growth inhibition zone of *S.mutans.* Adding propolis to orthodontic bracket adhesive will increase the antibacterial properties of the adhesive.

**2.** **Please add a statement or sentence about whether there are any similar studies that have been done before or not? Or is your study the first study to investigate this topic?**

Hatunoglu et al. (2014) has investigated that the addition of 25% and 50% Turkish propolis to glass ionomer cements increased antibacterial activity without modifying the mechanical properties of glass ionomer cements ^13^ but Hatunoglu et al. (2014) have not conducted time-related research. Research conducted by Megawati (2017) ^19^ shows that the addition of 25% concentration of propolis to resin modified glass ionomer cement does not affect shear strength ^19^. Further research on the effect of adding propolis concentrations of 0%, 15%, and 25% for 0, 15, and 30 days in resin modified glass ionomer cements on *Streptococcus mutans* growth inhibition zones has never been done. Therefore, our research was the first study to investigate the relationship between concentration and duration of propolis to the growth inhibition zone of *S.mutans*.

**3. Please add the critical or important issue within this study.**

The presence of fixed appliances in the oral cavity of orthodontic patients increases the microbial population, such as Streptococcus mutans known as bacteria in early or initial caries. Glass ionomer cement is known to release fluoride but the desired antibacterial effect is still lacking. ^13^ The antibacterial potential of propolis added to resin modified glass ionomer cement needs to be further investigated because orthodontic treatment takes place over a long period of time.

**4. Please add the reason why only S. mutans was examined in this study.**

*S. mutans* plays an important role in the etiology of caries. *S. mutans* is the first bacteria to form a colony and be the initiator of other bacteria to do the same thing. ^22^

**Material and Methods:**

**1.** **It would better if you add the detailed protocol or cite the protocol reference.**

For the preparation of propolis extract, it refers to the research conducted by Megawati (2017) ^19^.

For the preparation of resin modified glass ionomer cement containing propolis, it refers to to the research conducted by Megawati (2017) ^19 ^and Sodagar et al. (2017) ^20^. For the agar disk diffusion test, it refers to the research conducted by Yusuf (2016) ^21^.

**2. It would better if you add the confirmation of S. mutans colony or the detail of ATCC.**

The *Streptococcus mutans* used in our study was pure culture from the isolate laboratory of the Microbiology Faculty of Veterinary Medicine UGM, Yogyakarta, Indonesia.

**3.** **It would be better if you add more specific methods than agar disk diffusion test to examine the combination of propolis and GIC inhibit the *S. mutans.***

The diffusion method is most widely used to determine the sensitivity of bacteria to material because it is relatively simple and inexpensive. Unfortunately, it cannot determine the bactericidal and bacteriostatic properties of the drug. Hatunoglu (2014) has used a dilution method to determine the value of Minimum Inhibitory Concentration (MIC) of antibacterial material as the antibacterial capacity of glass ionomer cement with propolis ^13^. This has not been included in this project but it will surely be considered for our future work.

**4. In this study, the authors performed the Kruskal wallis test. It would be better if the authors also described the Levene's and Shapiro-Wilk test.**

Saphiro-Wilk normality test is conducted to determine the distribution of data and the Levene homogeneity test is carried out to determine the homogeneity of data variance. Data that is not normally distributed or not homogeneous are analyzed by the Kruskal-Wallis test and then the Mann-Whitney test was performed to determine differences in significance between treatment groups. The significance value of the normality and homogeneity test was (p> 0.05) while the significance value of the Kruskal Wallis and Mann-Whitney test was (p <0.05).

**Results:**

**1. It would be better to understand and attract the reader if you present the data in the diagrams.** **Please indicate whether the difference is significant or not in your graph/diagrams as well, using some symbols (asterisk).**

We will provide the data in the diagram with the new version of the article.

**2. Please add the results of the Levene's and Shapiro-Wilk tests**

The results of the Shapiro-Wilk test was data in this study that were not normally distributed (p = 0.012<0.05) and the results of the Levene test was the datas were homogeneous (p = 0.110>0.05).

**Discussion:**

Please mention the limitation(s) of this study in the discussion section.

Propolis is sufficient to be used as an antibacterial additive to resin modified glass ionomer cement, but it still needs further research aimed at the following:

1. get a combination of physical properties, propolis concentration, shear strength, and tensile strength that meet the optimal standard of orthodontic adhesive; and

2. increase stability and adequate propolis resistance.

We thank you again for this valuable insight.
